# Physical and Oxidative Stability of Uncoated and Chitosan-Coated Liposomes Containing Grape Seed Extract

**DOI:** 10.3390/pharmaceutics5030421

**Published:** 2013-08-20

**Authors:** Monika Gibis, Nina Rahn, Jochen Weiss

**Affiliations:** Department of Food Physics and Meat Science, Institute of Food Science and Biotechnology, University of Hohenheim, Garbenstr. 25, Stuttgart 70599, Germany; E-Mails: NinaRahn@gmx.de (N.R.); j.weiss@uni-hohenheim.de (J.W.)

**Keywords:** grape seed extract, polyphenols, chitosan, liposomes, coating, stability, lipid oxidation, hexanal

## Abstract

Polyphenol-rich grape seed extract (0.1 *w*/*w*%) was incorporated in liposomes (1 *w*/*w*% soy lecithin) by high pressure homogenization (22,500 psi) and coated with chitosan (0.1 *w*/*w*%). Primary liposomes and chitosan-coated secondary liposomes containing grape seed extract showed good physical stability during 98 days of storage. Most of the polyphenols were incorporated in the shell of the liposomes (85.4%), whereas only 7.6% of the polyphenols of grape seed extract were located in the interior of the liposomes. Coating with chitosan did not change the polyphenol content in the liposomes (86.6%). The uncoated liposomes without grape seed extract were highly prone to lipid oxidation. The cationic chitosan coating, however, improved the oxidative stability to some extent, due to its ability to repel pro-oxidant metals. Encapsulated grape seed extract showed high antioxidant activity in both primary and secondary liposomes, which may be attributed to its polyphenol content. In conclusion, the best chemical stability of liposomes can be achieved using a combination of grape seed extract and chitosan.

## 1. Introduction

The health benefits of grape seed extract (GSE; *Vitis vinifera*) have been demonstrated in numerous studies and can be attributed to its content of polyphenolic compounds. These polyphenols, besides gallic acid, consist mainly of flavonoids, including monomeric flavan-3-ols catechin, epicatechin, gallocatechin, epigallocatechin and epicatechin 3-*O*-gallate, as well as procyanidin dimers, trimers and more highly polymerized procyanidin [1]. The antioxidative activity of these substances protects the human body from premature aging and diseases, due to their free radical scavenging capability, metal chelating properties and reduction of hydroperoxide formation [2]. Furthermore, GSE exhibits antiproliferative activity and can inhibit the growth of colon cancer cells [3]. Chronic diseases, such as diabetes mellitus, cancer and cardiovascular diseases, are linked to an imbalance of antioxidants in the body. The “recommended dosage” of GSE to limit the risk of cardiovascular diseases varies from 100 to 300 mg/day [4].

Various studies have demonstrated that the activity of polyphenolic compounds may be retained or enhanced by incorporation into a carrier system [5]. Inclusion within the carrier materials results in protection of the active ingredients. Encapsulation can be performed by different techniques, such as spray-drying, spray chilling, extrusion coating, fluidized bed coating, coacervation, inclusion complexation, centrifugal extrusion, rotational suspension separation and liposome entrapment [5]. These techniques have been successfully used in the pharmaceutical, cosmetic, chemical and food industry. Liposome entrapment, in particular, is widely utilized for carriers of bioactive compounds [6].

Liposomes have been described as “closed, continuous bilayered structures composed mainly of lipid and/or phospholipid molecules” [7] that enclose an aqueous compartment, which allows the conveyance of molecules with different properties (hydrophilic, lipophilic and amphipathic) and characteristics, thus maintaining the stability and functionality of the encapsulated substances. Most polyphenolic compounds are incorporated into the bilayer membrane of the liposomes, whereas only a small part of them are located in the inner compartment of the liposomes [8,9]. Due to the ability to simulate the behavior of natural cell membranes, liposomes have been recognized by the pharmaceutical industry as a powerful tool in the treatment of diseases. They are used as drug delivery vesicles and for medical applications, such as in anticancer and gene therapy, vaccination and diagnostics [10]. Liposomes can entrap hydrophobic, as well as hydrophilic compounds within their structure. Encapsulation, therefore, is a potent technique that can be used to produce drug delivery systems with a controlled release of bioactive compounds, which can lead to an enhanced absorption of such molecules and to a more stable system with an extended shelf life [11,12,13]. Another approach to improve the oxidative stability of emulsions or liposomes is microencapsulation, where particles are coated with two or more interfacial membrane layers using the layer-by-layer deposition technique [8,14].

Coating with biopolymers, such as chitosan, has various advantages, because they exhibit a wide range of functionalities. Chitosan, for example, has antimicrobial and absorbing properties and is, therefore, used in medicine as a wound-dressing, because it does not exhibit any allergic effects [15]. Moreover, due to their high biocompatibility and biodegradability, chitosan nanoparticles have been increasingly used as drug carriers. As a new drug delivery system, they have attracted increasing attention for a wide range of applications, such as loading of protein and gene drugs, as well as anticancer chemical drugs. Such systems can be administered via different routes, including oral, nasal, intravenous and ocular. Chitosan nanoparticles have the advantage of slow and controlled drug release, which improves drug solubility and stability, enhances efficacy and reduces toxicity. Because of their small size, they are capable of passing through biological barriers *in vivo* (such as the blood-brain barrier) and delivering drugs to the lesion site [16].

Dehydration of the hydrophilic heads of lecithin molecules at an elevated temperature can alter their optimum curvature, which can lead to coalescence. A coating with chitosan may prevent this type of coalescence by inhibiting the close contact of the vesicles. Due to the higher magnitude of the ζ-potential in the chitosan-coated liposomes, the electrostatic repulsion between the droplets is larger than in the primary liposomes, and the thickness of the membrane is enlarged [17]. This leads to vesicles that are capable of generating strong electrostatic repulsive forces. Thus, pro-oxidant metals are repelled from the vesicle surface, and the lipid oxidation rate decreases [18]. *In vitro* digestion experiments have shown a slower release of fatty acids from multilayer emulsions with chitosan coating, thus further reducing digestion [19]. Therefore, the addition of chitosan may create an emulsion delivery system that will pass through the stomach and small intestine and, then, release its bioactive lipids into the lower portion of the gastrointestinal tract when the polysaccharides are digested via bacterial fermentation [19,20].

In the present investigation, we hypothesized that coating the GSE-rich liposomes with chitosan may improve their physical and chemical stability against lipid oxidation. To test our hypothesis, the primary and secondary liposomes (with or without encapsulated GSE) coated with chitosan were prepared by the layer-by-layer deposition method. In addition, we postulated that encapsulation efficacy may be possible, because the encapsulated polyphenols are mostly incorporated into the membrane of the liposomes. All the liposomes were tested for their physical and oxidative stability.

## 2. Experimental Section

### 2.1. Material

GSE was provided by Plantextrakt GmbH & Co. KG (Martin Bauer Group, Vestenbergsgreuth, Germany). The spray-dried GSE contained 40% of polyphenols (calculated as anhydrous gallic acid), of which 30% were procyanidins (calculated as cyanidin chloride). The soy lecithin (Lipoid S75) was obtained from Lipoid AG (Ludwigshafen, Germany). It contained 69.3% phosphatidylcholine, 9.8% phosphatidylethanolamine and 2.1%, lysophosphatidylcholine and had a fatty acid composition of palmitic (17%–20%), stearic (2%–5%), oleic (8%–12%), linoleic (58%–65%) and linolenic (4%–6%) acid, according to the company’s specifications. Low molecular weight chitosan (purity > 78%, degree of deacetylation, 79%, viscosity, 103 cP), hexanal, acetic acid, anhydrous sodium acetate, Folin-Ciocalteu reagent (1.9–2.1 N) and Triton X-100 (analytical grade) were purchased from Sigma Aldrich (St. Louis, MO, USA). Deionized, distilled water was used throughout the experiments.

### 2.2. Preparation of Extract Solution

An acetate buffer (0.25 mol/L, pH 3.7 ± 0.1) was prepared with 1.421 g/L acetic acid and 0.181 g/L anhydrous sodium acetate. The extract solution was prepared by dissolving 0.11 *w*/*w*% GSE in acetate buffer. The extract solution was stirred for 1 h and then filtrated using folded cellulose filters (MN 616, Macherey & Nagel, Düren, Germany). The main monomeric polyphenols of the filtrated GSE were quantified, and the dimeric polyphenols were only identified by (High Performance Liquid Chromatography Elektrospray-Ionisation-Tandem Mass Spectrometry) HPLC-ESI-MS/MS [21]. The composition was as follows: procyanidin B1 (19.3 mg/g), (+)-catechin (19.1 mg/g), (−)-epicatechin (10.3 mg/g), procyanidin B2 (5.3 mg/g), gallic acid (2.6 mg/g), (−)-epicatechin gallate (2.1 mg/g), and galloylated dimeric procyanidin and dimeric procyanidins [22].

### 2.3. Preparation of Primary Liposomes

Two different types of primary liposomes were prepared: (i) a 1.1 *w*/*w*% lecithin solution was prepared by dissolving 5 g lecithin in 500 g acetate buffer; and (ii) a 1.1 *w*/*w*% lecithin solution containing GSE was prepared by dissolving 5 g lecithin in 500 g GSE solution (0.11 *w*/*w*%). Both solutions were stirred at room temperature overnight on a magnetic stirrer. Both solutions were then passed five times through a high pressure homogenizer (Microfluidics International Cooperation, Newton, MA, USA) at 22,500 psi. Both primary liposomes were diluted with acetate buffer (9:10) to the same concentration as the secondary liposomes and stored in the dark at room temperature.

### 2.4. Preparation of Secondary Chitosan-Coated Liposomes

A chitosan solution (1 *w*/*w*%) was prepared by dissolving chitosan in acetate buffer (0.25 mol/L, pH 3.7) on a magnetic stirrer at room temperature overnight. Two different types of secondary liposomes were prepared using the following primary liposomes: (i) a 1.1 *w*/*w*% lecithin solution was analogously prepared as the primary liposomes in acetate buffer; and (ii) a 1.1 *w*/*w*% lecithin solution containing GSE was prepared in GSE solution (0.11 *w*/*w*%). To obtain secondary liposomes, 1 mL chitosan solution was pipetted into a test-tube, and 9 mL liposomal solution (1.1 *w*/*w*%) was added while the test-tube was vortexed. Secondary liposomes with GSE were prepared in the same way. Both primary and secondary liposomes had the same concentrations of GSE and lecithin after preparation. The secondary liposomes were stored in the dark at room temperature.

### 2.5. Particle Size Determination

Dynamic light-scattering was performed using a dynamic light-scattering instrument (Nano ZS, Malvern Instruments, Malvern, UK). The Zetasizer detects back-scattered laser-light at a scattering angle of 173° at 25 °C. A solvent refractive index of 1.335 was used. The instrument measures the coherence of scattering patterns as a function of time. The decay of coherence is then converted to apparent particle sizes and distributions via the software, which relies on Mie theory calculations. Primary and secondary liposomes were diluted to a lecithin concentration of approximately 0.01 *w*/*v*% with acetate buffer to prevent multiple scattering effects. This technique is based on the scattering of light by moving particles due to Brownian motion in a liquid. The size is then calculated from the diffusion constant using the Einstein equation. The instrument reports the mean particle diameter (z-average) and the polydispersity index (PDI), ranging from 0 (monodisperse) to 1 (polydisperse); a value >0.5 indicated a broad particle distribution.

### 2.6. ζ-Potential Measurements

Liposome solutions were diluted to a lecithin concentration of approximately 0.01 *w*/*v*%. The samples were then loaded into a cuvette of a particle electrophoresis instrument (Nano ZS, Malvern Instruments, Malvern, UK), and the ζ-potential was determined by measuring the direction and velocity that the liposomes moved in the electric field applied and calculated using the Smoluchowski equation. The measurements of ζ-potential were calculated as the average and standard deviation of measurements made from two freshly prepared samples, with two readings made per sample.

### 2.7. Gas Chromatography (GC)

A CP-3800 gas chromatograph (Varian Instrument Group, Walnut Creek, CA, USA) equipped with Varian Galaxie software and with a QHSS^®^40 headspace sampler (QUMA Elektronik & Analytic GmbH, Wuppertal, Germany) using a 1 mL sample loop was used to determine the secondary oxidation product of lecithin and hexanal, in primary and secondary liposomes with and without GSE. Hexanal was determined according to the method of Romeu-Nadal *et al*. [23] with some modification. Liposome samples (1 mL) were placed into a 20 mL HSS headspace vial sealed with polytetrafluoroethylene (PTFE) septa (QUMA Elektronik & Analytic GmbH, Wuppertal, Germany). Samples were equilibrated for 15 min at 55 °C in the autosampler heating oven and, then, transferred to gas chromatography (GC) with the gas-sampling valve (90 °C) and tube temperature set at 150 °C. The chromatographic separation of volatile aldehydes was performed on a fused-silica capillary column J&W HP-FFAP (30 m, inside diameter 0.32 mm, 0.25 µm) purchased from Agilent Technologies (Waldbronn, Germany). Hydrogen was used as the carrier gas (split ratio 1:50), nitrogen as the make-up gas and synthetic air and hydrogen as the gas for the flame ionization detector. The oven program was set to 60 °C for 3 min and, then, heated at 15 °C/min for 15 min until 230 °C was reached. The flame ionization detector and the GC injector both had a temperature of 250 °C. Hexanal concentrations were determined using a standard curve made from a stock solution of hexanal (1148.30 nmol/mL). A standard solution containing different amounts of hexanal (concentrations: 38.28, 76.55, 153.11, 382.77, 765.53 and 1148.30 nmol/mL) was used for calibration. All tests were performed in triplicate.

### 2.8. Folin-Ciocalteu Assay

A Folin-Ciocalteu assay was used to determine the content of phenolic compounds in the different liposomes containing GSE [24]. This reagent reacts with the phenolic compounds in the sample, which were detected at 720 nm using an HP UV-Vis 8453 spectrophotometer (Hewlett Packard, Waldbronn, Germany). Both the samples and the Folin-Ciocalteu reagent were diluted 1:10 in water for the determination. An amount of 1 mL of the diluted sample was mixed with 5 mL of diluted Folin-Ciocalteu reagent in a test-tube, stirred with a vortexer and left for 3 min. Then, 4 mL of sodium carbonate solution (prepared by dissolving 7.5 g sodium carbonate in a 100 mL flask with water) was added, and the test-tube was again vortexed. In the blank sample, 1 mL of water was used instead of the liposome sample. Liposomes with GSE were also measured as sample blanks without adding Folin-Ciocalteu reagent to assess and correct the intensity of extinction caused by absorption of liposomes and chitosan at the wavelength measured. The total amount of phenolic compounds was determined using an external standard made from gallic acid (stock solution 500 mg/L in ethanol). The concentrations of gallic acid for the calibration curve were 5, 10, 20, 30, 40 and 50 mg of gallic acid/L in water and prepared analogously as the samples. All measurements were carried out twice. It should be noted that the results may not reflect the exact amount of phenolic compounds in the samples, because other reducing compounds beside the phenolic compounds can react with the Folin-Ciocalteu reagent.

Primary and secondary liposomes were filtrated by Sephadex-gel filtration. An amount of 0.5 *w*/*w*% Sephadex G50 was dissolved in deionized water. Syringes (6 mL) were filled with hydrated Sephadex G50 particles until a layer of about 3 cm of gel had been formed, and 1.5 mL of acetate buffer (pH 3.7, 0.25 mol/L) was added on top of the gels. The Sephadex G50 column was then centrifuged at 3000 rpm for 10 min (Sepatech Biofuge 28 RS, Heraeus, Hanau, Germany). The liposomes were gel filtrated analogously. The samples were then treated with Triton X-100 to break open the liposomes. Extract that did not interact with the liposomes can be removed by Sephadex-gel filtration. The liposomes are broken with the addition of Triton X-100, and the total amount of phenolic compounds in the inner compartment and in the membrane of the liposomes can be measured.

### 2.9. Statistical Method

All measurements were repeated at least three times using duplicate samples. Means and standard deviations were calculated using Excel (Microsoft, Redmond, WA, USA). The data of particle diameter and hexanal were tested for normal distribution using the Shapiro-Wilk test. When the data was normally distributed, the values were analyzed by a variance analysis using the GLM (general linear model) procedure and the Tukey test (α = 0.05) with the version 9.3 SAS program (SAS Institute INC., Cary, NC, USA). When the data was not normally distributed, a non-parametric Kruskal-Wallis test was used to determine differences between the different liposomal systems at the same storage time.

## 3. Results and Discussion

### 3.1. Optical Appearance of Liposomal Systems

**Figure 1:**
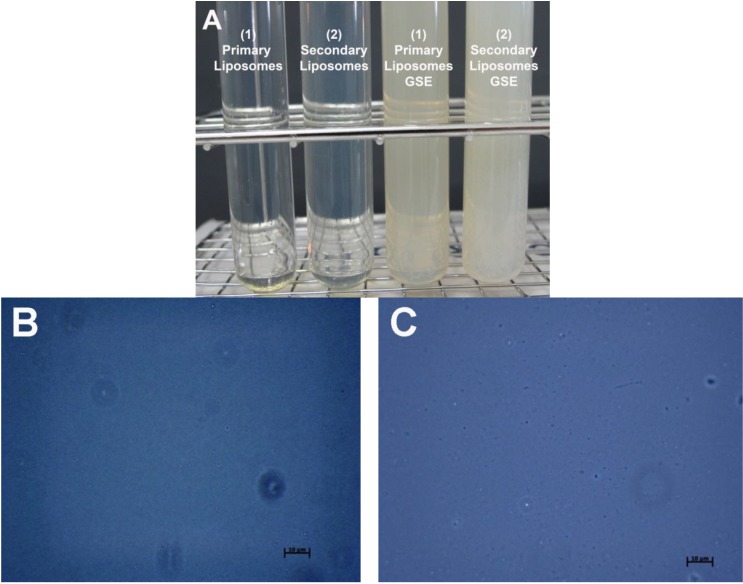
(**A**) Photographic images of (1) primary liposomes (1 *w*/*w*% lecithin) and (2) chitosan-coated secondary liposomes with and without incorporated grape seed extract (GSE) (0.1 *w*/*w*% GSE, 1 *w*/*w*% lecithin, 0.1 *w*/*w*% chitosan); (**B**) optical microscope images of primary liposomes containing GSE and (**C**) of secondary liposomes containing GSE. Magnification: 1000× (scale bar: 10 μm).

The visible appearance after preparation is illustrated in Figure 1A. Both primary and chitosan-coated secondary liposomes without GSE were clear and optically transparent. By comparison, the liposomal systems containing GSE had both an opaque and turbid appearance. The primary and secondary liposomes with GSE showed only a slight difference in their turbidity. The turbidity is caused by the larger size of liposomes containing GSE scattering more light. The turbid solutions were then analyzed using optical microscopy to determine if any aggregation had occurred. The optical microscope only detects particles with a z-average mean diameter >500 nm. No aggregation was detected in the turbid liposomal solutions (Figure 1B,C). This is in accordance with previous results of optical appearance in secondary liposomes coated with a biopolymer layer of chitosan [8].

### 3.2. Effect of Encapsulation of Grape Seed Extract and Coating with Chitosan on Particle Diameter and ζ-Potential of Liposomes

**Figure 2:**
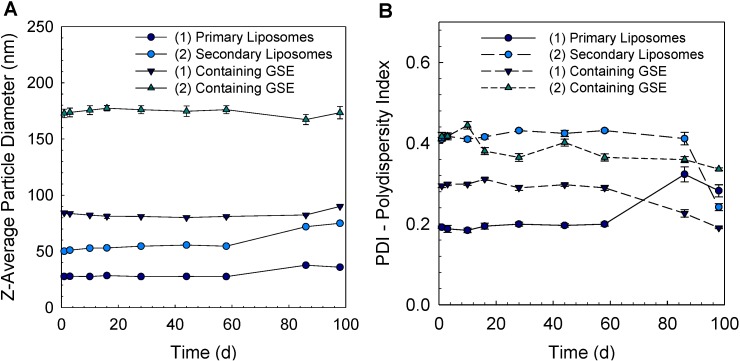
(**A**) Z-average diameter and (**B**) polydispersity index (PDI) of (1) primary and (2) secondary liposomes with and without incorporation of GSE (1 *w*/*w*% lecithin, 0.1 *w*/*w*% chitosan, 0.1 *w*/*w*% GSE) during storage for 98 days.

The physical stability of liposomes was investigated by measuring their particle size (z-average diameter) during 98 days (Figure 2). Uncoated “empty” liposomes without GSE had the smallest particle size (28 nm); however, when GSE was incorporated into the primary liposome, its particle size increased three-fold (84 nm) (Figure 2A). On the other hand, secondary liposomes coated with chitosan had a particle size of 50 nm. Encapsulation of GSE into secondary liposomes increased the particle size to 173 nm. The particle size of the primary liposomes with or without GSE did not change during 58 days (*p* > 0.05). After 58 days, the particle diameter of primary liposomes without GSE had slightly increased to 36 nm and, with GSE, to 90 nm (*p* < 0.001) after 86 days (Figure 2A). The secondary liposomes without extract were stable for a time period of 58 days. No significant differences between the measurements were identified during this storage time. After 58 days, the z-average particle diameter increased to 75 nm. The z-average particle diameter of the secondary liposomes containing GSE showed no significant changes in the z-average particle diameter (*p* > 0.05) over the total storage period of 98 days.

The polydispersity index (PDI) is regularly used as an indicator of the broadness of the particle size distribution. Primary liposomes had the smallest PDI (0.2), indicating that the solution was monodispersed, whereas the other liposome systems were polydispersed (Figure 2B). PDI values of the various liposomal systems showed no changes for 58 days. After 58 days, the primary liposomes without GSE had a broad particle size distribution, due to the oxidative degradation of the unsaturated fatty acids in phospholipids.

**Figure 3:**
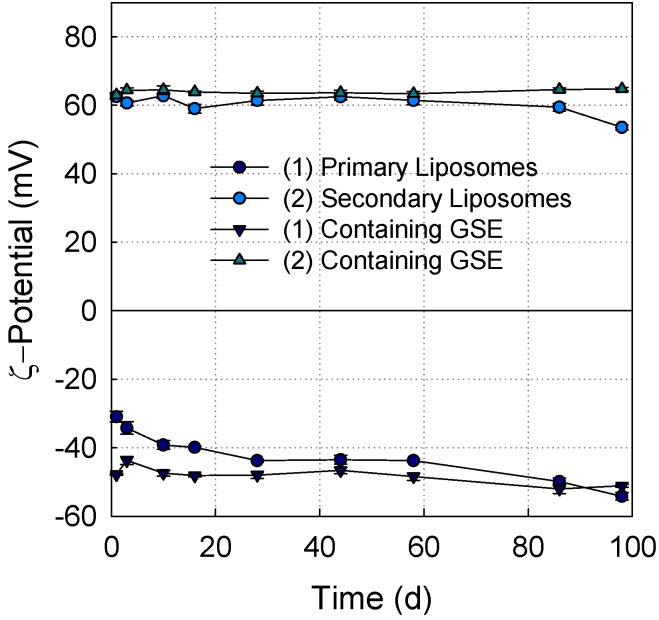
ζ-potential of (1) primary liposomes and (2) secondary liposomes with and without GSE (1 *w*/*w*% lecithin, 0.1 *w*/*w*% chitosan, 0.1 *w*/*w*% GSE) during storage for 98 days.

ζ-potential is a useful indicator of the surface charge of the particles, which governs the repulsive colloidal interactions, as well as the physical stability of the coated liposomal systems [25]. The ζ-potential of the liposomes is shown in Figure 3. By coating the liposomes with chitosan, the ζ-potential changed from −49 mV to +63 mV (Figure 3). Uncoated liposomes with GSE had a lower ζ-potential (−48 mV) than uncoated liposomes without GSE (−42 mV). This may be due to the negatively charged carboxyl group of gallic acid in GSE [8]. Secondary liposomes with or without chitosan coating had a similar ζ-potential (Figure 3). The chitosan coating increased the repulsive interaction between the secondary liposomes, thus preventing the particles from flocculating [25]. No significant changes in the ζ-potential of the liposomes could be detected during storage. In summary, all liposomes were physically stable during the 98 days of storage, as indicated by the ζ-potential (Figure 3) and the particle diameter (Figure 2) measurements.

### 3.3. Determination of Total Phenolic Compounds and Entrapment Efficiency

The content of total phenolic compounds in all systems containing GSE was determined with a Folin-Ciocalteu reagent. The results are shown in Table 1. The entrapment efficiency (EE) is an important parameter in the evaluation of liposomes containing GSE. The values of primary and secondary liposomes are calculated with Equation 1:

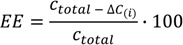
(1)
where *c_total_* is the concentration of total polyphenols of GSE and Δ*c_(i)_* is the difference between the amount of total polyphenols and the polyphenols of primary or secondary liposomes after gel filtration and treatment with the surfactant, Triton X-100.

**Table 1:** Concentration of total polyphenolic compounds (mg gallic acid/L) of GSE alone and incorporated into different liposomal systems measured by the Folin-Ciocalteu assay.

Sample type	Concentration of phenolic compounds mg gallic acid/L	Difference between sample type phenolic compounds mg gallic acid/L
GSE	406 ± 3	na
GSE ^a^	1 ± 22	na
Primary liposomes containing GSE ^b^	381 ± 17	na
*Free polyphenols* ^c^	na	25 ± 20 ^c^
Primary liposomes with GSE ^a^	350 ± 1	na
*Polyphenols in the core* ^d^	na	31 ± 20 ^d^
Secondary liposomes with GSE ^a^	292 ± 1	na
*Polyphenols in the core* ^e^	na	63 ± 12 ^e^
Secondary liposomes containing chitosan and GSE ^b^	355 ± 10	na

na, not analyzed; ^a^ Sephadex-gel filtration was performed to remove the non-encapsulated polyphenols; ^b^ Sephadex-gel filtration was performed to remove the non-encapsulated polyphenols, and Triton X-100 treatment was used to break down the liposomes to calculate the concentration of total polyphenols in their inner core and membrane; ^c^ non-incorporated GSE polyphenols (free polyphenols) calculated by *Δc=c_(a)_* − *c_(c)_*; ^d^ GSE polyphenols in the liposome core calculated by *Δc=c_(c)_* − *c_(d)_*; ^e^ GSE polyphenols in the liposome core calculated by *Δc=c_(e)_* − *c_(f)_*.

The results showed that 92.2% of the GSE was incorporated into the shell of the primary liposomes (Table 1). The entrapment efficiency in the secondary liposomes was 87% (Table 1). Only 7.6% of the GSE was located in the interior of the liposomes. After coating with chitosan, 17.7% of polyphenols were still detectable in the inner core of the liposomes (Table 1). This may be because the chitosan layer is not dense enough, and thus, the Folin-Ciocalteu reagent can still interact with the polyphenols of the extract. The entrapment efficiency of the polyphenolic compounds of GSE was found to be 83.5% in an earlier study [8], which was slightly lower than in the present study.

### 3.4. Lipid Oxidation of Liposomes

**Figure 4:**
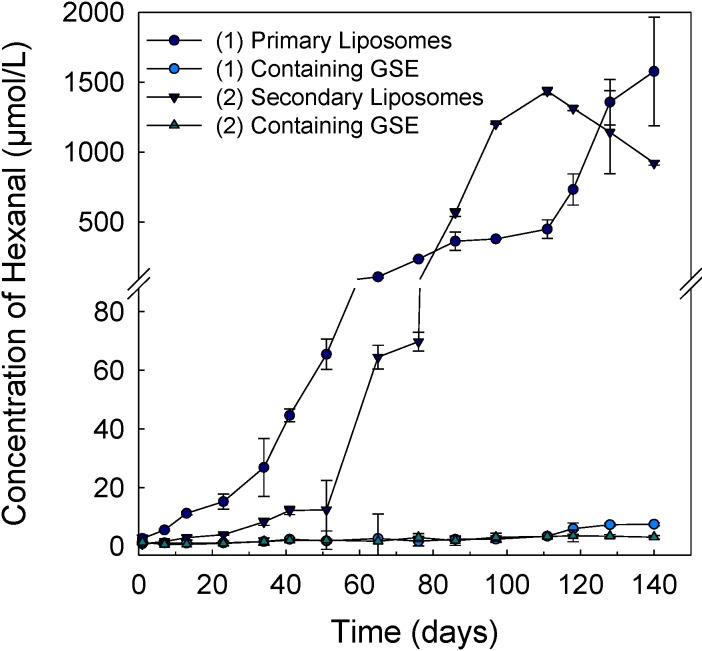
Concentration of hexanal in primary (1) and secondary liposomes (2) with or without encapsulated GSE during a time period of 140 days in the dark at 25 °C.

The lipid oxidation of different liposomes was measured during 140 days in the dark at 25 °C (Figure 4). Hexanal was used as an indicator for lipid oxidation, because hexanal is a specific volatile oxidation product of *n*−6 polyunsaturated fatty acids, such as linoleic acid, which is the main fatty acid in the soy lecithin used. The results show that the formation of hexanal increased rapidly in the uncoated liposomes (without GSE) after seven days of storage (Figure 4). The chitosan-coated liposomes without GSE had a lag phase of 50 days before the lipid oxidation propagated. This indicates that the chitosan coating was able to inhibit the formation of hexanal to some extent. The cationic chitosan on the liposome vesicles may repel the pro-oxidant metals from the liposome surface and, thus, inhibit metal-induced lipid oxidation [17]. However, as shown in Figure 4, the formation of hexanal increased rapidly in chitosan-coated liposome after 50 days. By contrast, primary and secondary liposomes containing GSE were effective at inhibiting the formation of hexanal (Figure 4). No statistical differences were found between the primary and secondary liposomes containing GSE up to 118 days. After 118 days, the secondary liposomes with entrapped GSE showed a significantly lower (*p* < 0.05) concentration of hexanal in comparison to the primary liposomes containing GSE. Thus, the antioxidant activity can be attributed to the antioxidant activity of polyphenols in GSE. This is in accordance with a previous study showing a similar physical and oxidative stability of chitosan-coated liposomes with incorporated rosmarinate esters [17]. Their results suggested that combining the inclusion of appropriate antioxidants, such as rosmarinic acid, and the deposition of a chitosan coating onto the surface of liposomes may significantly increase the oxidative and physical stability of liposomes [17]. This additive or synergistic effect of polyphenols in GSE and chitosan coating may be caused by the chelating of pro-oxidants, such as metals, and the high antioxidative activity of GSE polyphenols, as well as the cationic surface charge of chitosan. As chitosan forms a charged barrier to inhibit the contact of metals ions with the phospholipid surface, this also leads to reduced interactions of metals with primary oxidation products, such as lipid hydroperoxides [17].

This inhibits the formation of free radicals and, thus, prolongs the oxidative degradation of phospholipids, as was observed in the uncoated primary liposomes without extract (Figure 4). Therefore, incorporation of both GSE polyphenols and chitosan coating can improve the oxidative stability of liposomes.

## 4. Conclusions

All liposomes showed good physical stability, confirmed by the particle diameter and the ζ-potential measurements during six weeks of storage. Most polyphenolic compounds were incorporated into the membrane of the liposomes. Only 7.6% of the GSE was located in the core of the liposomes. However, the chitosan coating did not fully prevent the reaction of GSE polyphenols with the Folin-Ciocalteu reagent, possibly because the chitosan layer was not dense enough to inhibit the reaction. Primary and secondary liposomes with GSE showed good antioxidant activity against lipid oxidation. However, the chitosan coating alone was also shown to prolong the lipid oxidation to some extent. In conclusion, the best oxidative stability in liposomes can be achieved with a combination of GSE and chitosan. Future studies should focus on investigating the release of GSE in chitosan-layered liposomes and the biological and technological functionality of the encapsulated GSE, including interactions of the encapsulation system with other ingredients. Additionally, the mechanism of the binding processes and the precise nature of the developing membrane should be investigated.
